# Predicting HIV-1 broadly neutralizing antibody epitope networks using neutralization titers and a novel computational method

**DOI:** 10.1186/1471-2105-15-77

**Published:** 2014-03-19

**Authors:** Mark C Evans, Pham Phung, Agnes C Paquet, Anvi Parikh, Christos J Petropoulos, Terri Wrin, Mojgan Haddad

**Affiliations:** 1 Monogram Biosciences Inc., 345 Oyster Point Blvd., South San Francisco, CA 94080, USA

**Keywords:** HIV-1 antibody, Thick patch analysis, Bioinformatics algorithms, Boosting algorithm, Machine learning, Neutralization, *in-silico* epitope mapping, Epitope networks, Structural mapping, Sequence and structure analysis

## Abstract

**Background:**

Recent efforts in HIV-1 vaccine design have focused on immunogens that evoke potent neutralizing antibody responses to a broad spectrum of viruses circulating worldwide. However, the development of effective vaccines will depend on the identification and characterization of the neutralizing antibodies and their epitopes. We developed bioinformatics methods to predict epitope networks and antigenic determinants using structural information, as well as corresponding genotypes and phenotypes generated by a highly sensitive and reproducible neutralization assay.

282 clonal envelope sequences from a multiclade panel of HIV-1 viruses were tested in viral neutralization assays with an array of broadly neutralizing monoclonal antibodies (mAbs: b12, PG9,16, PGT121 - 128, PGT130 - 131, PGT135 - 137, PGT141 - 145, and PGV04). We correlated IC_50_ titers with the envelope sequences, and used this information to predict antibody epitope networks. Structural patches were defined as amino acid groups based on solvent-accessibility, radius, atomic depth, and interaction networks within 3D envelope models. We applied a boosted algorithm consisting of multiple machine-learning and statistical models to evaluate these patches as possible antibody epitope regions, evidenced by strong correlations with the neutralization response for each antibody.

**Results:**

We identified patch clusters with significant correlation to IC_50_ titers as sites that impact neutralization sensitivity and therefore are potentially part of the antibody binding sites. Predicted epitope networks were mostly located within the variable loops of the envelope glycoprotein (gp120), particularly in V1/V2. Site-directed mutagenesis experiments involving residues identified as epitope networks across multiple mAbs confirmed association of these residues with loss or gain of neutralization sensitivity.

**Conclusions:**

Computational methods were implemented to rapidly survey protein structures and predict epitope networks associated with response to individual monoclonal antibodies, which resulted in the identification and deeper understanding of immunological hotspots targeted by broadly neutralizing HIV-1 antibodies.

## Background

To date, the design of an effective vaccine against Human Immunodeficiency Virus-1 (HIV-1) remains a challenge and has failed to produce broad and effective neutralization responses [1-8]. The design of protective immunogens is especially challenging due to the high viral escape rate from immune control [9-11]. Ongoing HIV-1 vaccine research efforts include finding and characterizing broadly neutralizing antibodies (nAbs), and the epitopes they target [12,13]. Identification of the antigenic targets of nAbs along with mapping the immunologically important residues of known epitopes that affect neutralization is therefore a major goal of current HIV-1 vaccine research. The HIV-1 envelope is highly variable, and as a consequence, identification of key residues that affect neutralization can be complex. In some instances, lack of neutralization can be explained by amino acid changes in the known epitopes, but in other cases epitope conservation does not ensure neutralization [14]. In addition, many regions outside of the known epitopes have been shown to affect neutralization sensitivity [15]. The aim of this study is to develop a computational method for discovering and evaluating “epitope networks” that we define here as groups of interacting and variable residues that affect antibody binding.

A key element in successful immune response is the interaction between foreign antigens and antibodies produced by the B-cells. The ability to identify and characterize epitopes on antigen surfaces is important for vaccine design, the development of antibody therapeutics, and immunodiagnostic tests. In the last decade, significant effort has been invested to understand the nature and characteristics of linear epitopes with the goal of developing reliable methods for predicting them. Many tools of varying utility were produced and have been reviewed [16]. One significant outcome was the realization that there is no single measurable feature about protein-protein interactions that is able to reliably predict antibody binding sites. More recently, studies have been performed to address conformational epitope identification and prediction which resulted in several useful tools. These have been reviewed in detail by El-Manzalawy [17]. In general, existing methods for predicting conformational B-cell epitopes can be grouped into three categories: those that rely upon antigen protein structure alone [18-20], those that use antigen structure in combination with the antibody peptide sequence [21,22] and those that map peptide “mimics”, mimotopes, derived from random peptide libraries to the antigen structure’s surface [23-26].

In this paper, we describe a novel method that utilizes the antigen protein structure together with neutralization titers measured by Monogram Biosciences’ neutralization assay [9] to predict functional B-cell epitope networks and key protein-protein interacting residues. Data generated from Monogram’s neutralization assay has been previously used by researchers utilizing alanine scanning and various other lab techniques to characterize monoclonal antibodies (mAbs) [3,4,14,27]. Our goal was to develop a rigorous computational method that incorporates neutralization sensitivity data from a panel of naturally occurring viruses, in combination with sequence and protein structure information, and applies an ensemble of data mining techniques to enable rapid and accurate prediction of antibody epitope networks. We aimed to investigate residues that can interact with antibodies as a network, and in a structurally meaningful way. We therefore evaluated envelope sequences grouped into patches of amino acid sites. These patches were then examined to discover networks of variable residues that significantly impact neutralization sensitivity.

Patch analysis has been previously suggested and performed to predict protein-protein interaction sites [28,29]. To identify potential HIV-1 antibody epitope network residues on the antigen surface, we started with the common concept of a surface patch, as previously described [30]. Surface patches are typically defined by determining the accessible surface area (ASA) of each residue and taking those positioned above a certain threshold. These values can be calculated by a number of tools (DSSP [31] and NACCESS [32] for example) which use tables of maximum solvent accessibility for each amino acid in a Gly-X-Gly or Ala-X-Ala tripeptide configuration in solution. However, work by Singh et al. [33] in which they examined the surface accessibility of over 18,000 structures in the protein data bank (PDB), showed that observed surface accessibility often differed significantly from the values found in traditionally used tables [34,35] and that occasionally the use of these values in a real-world calculation produced a result where an amino acid had a predicted ASA value of more than 100%. To address this, Singh et al. developed a table of highest observed accessibility (HOA) values and suggested normalizing ASA by HOA to obtain a more meaningful relative surface accessibility (RSA) [33].

The concept of residue interaction networks (RIN) has been discussed recently in several publications [36,37]. The principle is based upon the fact that proteins are not rigid bodies, as in a crystal structure, but rather that they are for the most part flexible and can change in response to their immediate environment. Likewise, in a protein-protein interaction, the orientation of an interacting residue can be influenced by other, buried, residues that are immediately below it as well as the residues located deeper underneath to some extent. Residue depth has been correlated with several protein properties, such as stability, and conservation of amino acids and their types [38]. Pintar et al. [39] defined atom depth as the distance in angstroms (Å) of a non-hydrogen buried atom from its closest solvent accessible protein neighbor. Using this definition, the residue depth (DPR) of surface residues is defined as 0, and >0 for all those that are buried. In an examination of structures in the PDB, it was found that buried atoms tend to cluster into discrete layers [39]. The first inner layer has a maximum DPR of ≈ 1.50 Å, with a second inner layer having a maximum DPR of ≈ 2.50 Å. It is then possible to use the mean residue DPR to construct “thick” surface patches in an attempt to capture key residues from the RIN that are important in defining a functional epitope.

In this study, we determined relative solvent accessibility according to the method of Singh et al. [33] when examining the structural information. Additionally, we took the RIN data into account by exploring thick patches with several thresholds for DPR. We generated structural patches considering the complete information according to these parameters, and systematically tested each patch for its ability to determine neutralization response. Our goal was to identify epitope networks which correspond to immunogenic regions recognized by nAbs.

We were able to build a bioinformatics process, called “thick patch analysis” (THIPA), that combines viral sequence and structural information as well as neutralization titers against viruses with a range of HIV-1 subtypes, and applies an ensemble of machine learning and statistical models to predict antibody epitope networks. In this paper, we describe the computational method, and the predicted epitope networks for 21 HIV-1 mAbs, as well as the results from the lab experiments we performed in order to validate the algorithm.

## Methods

### Neutralization assay

A recombinant-virus assay involving a single round of virus infection was used to measure neutralization [9]. Briefly, the HIV-1 *env* libraries present in patient plasma or cell culture supernatant were amplified from the source material and cloned into the *env* expression test vector. The cloned env libraries were co-transfected with an *env*-deleted genomic vector containing a luciferase reporter and the resulting “pseudotyped” viruses were pre-incubated for 1 hour with serial dilutions of monoclonal antibodies (MAb) [3,4] and then used to infect U87 cells that express CD4 plus the CCR5 and CXCR4 co-receptors (U87/CD4/CCR5/CXCR4). Neutralizing activity was expressed as the percent inhibition of viral replication (luciferase activity) at each antibody dilution or concentration compared to a control culture without antibody. The 50% inhibition concentration (IC_50_) was determined and expressed as the antibody concentration conferring 50% inhibition (Additional file 1: Figure S1). Since the *env* libraries have mixed nucleotides throughout the gp160 sequence reflecting the sequence diversity within the viral quasispecies, individual clones were selected so that unambiguous sequence from a single gp160 vector clone was used in the analysis. For clonal selection, the pooled viral DNA was diluted and used to transform competent *E. coli*. For each patient sample, multiple colonies were picked and screened by a rapid single replication cycle assay. Multiple clones, totaling more than 850, were screened for sensitivity to nAbs using 3 broadly neutralizing MAbs (b12, 2 F5 and PG9) that act on different regions of *env*. Clones were selected for this study based on either being similar to the neutralization sensitivity pattern with the mAbs, co-receptor tropism and infectivity of the parental *env* library or because they differed dramatically from the phenotype of the library in any of the cited characteristics.

### Data set

We obtained a panel containing 282 clonal viruses collected from 175 unrelated patients who submitted samples for commercial patient testing at Monogram Biosciences. The 282 clones are of diverse subtypes and recombinants: 21 subtype A’s, 24 AE’s, 19 AG’s, 55 B’s, 3 BF’s, 32 C’s, 55 D’s, 24 F1’s, 41 G’s, 3 H’s, and 5 subtype J’s. They also differed in co-receptor tropism with 205 R5-only clones, and 77 non-R5 viruses. All clones were tested in the neutralization assay, described above, and IC_50_ titers were obtained for an array of 21 neutralizing monoclonal antibodies (b12, PG9,16, PGT121 - 128, PGT130 - 131, PGT135 - 137, PGT141 - 145, and PGV04) [40,41].

### Virus sequencing and sequence analysis

**Figure 1:**
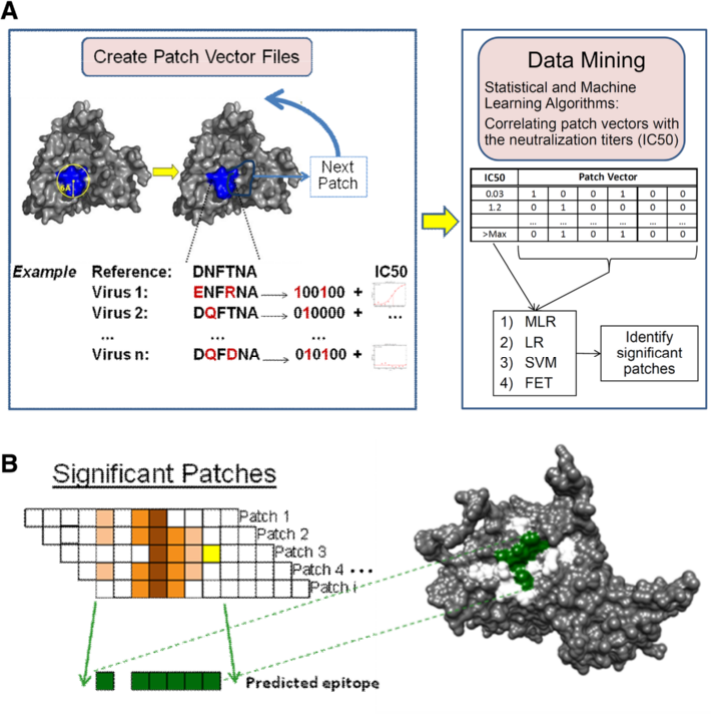
**THIPA (Thick Patch Analysis): An Automated Epitope Network Prediction Pipeline. A)** Patch vector files are generated using structural, genotypic, and phenotypic data. Subsequently, patches are analyzed by multiple machine learning and statistical methods, and patches identified as significantly associated with neutralization by all models are selected. **B)** Significant patches are then aligned by position to identify residue clusters that are frequently recognized as significant. The overlapping residues identified more frequently than average are reported as predicted epitope networks and are modeled into the 3D structure (highlighted in this cartoon example in green).

Clonal sequences were obtained for this virus panel. A set of 12 primers was used to generate overlapping and redundant sequences from both DNA strands. The amino acid sequences were aligned to the gp160 sequence of the laboratory reference strain HXB2 (GenBank accession number K03455), and amino acid positions were numbered through the alignment with the reference sequence. HXB2 is commonly used for alignment and numbering by researchers studying HIV-1 envelope sequences [14,42,43]. Sequence alignments were performed using ClustalW2 [44] and key sequence regions were identified by the profile Hidden Markov Model (HMM) software *hmmscan*[45]. A binary representation of each sequence was obtained by comparison to HXB2: positions with amino acids identical to HXB2 were coded as 0 and amino acids different from HXB2 were coded as 1 (Figure 1A). Insertions and deletions were reported as 1 at the HXB2 position preceding the gap or insertion.

### THIPA process overview

The process of predicting antibody epitope networks is streamlined into an analysis pipeline called “thick patch analysis” (THIPA) (Figure 1). The THIPA process is performed as follows: first, patch vectors are generated based on the structural model and genotype of the antigen; as a second step, statistical and machine learning analyses are performed to identify significant association of patch compositions and IC50; third, patches significantly correlating with IC50 are aligned by position; finally, the most frequently occurring residue positions within all significant patches are mapped onto the structure and identified as predicted epitope network for the antibody. These steps are described in details in the following sections.

### Patch definition

Surface patches are determined by taking a suitable antigen protein structure and for any given surface residue, determining which additional residues within a given radius from the central residue meet the appropriate RSA and DPR criteria. The residues in a surface patch are frequently not sequential, but represent a conformation-dependant surface.

To be able to successfully identify all solvent accessible residues on the gp120 antigen surface, it is important to identify a suitable protein structure. Of the more than 24 x-ray structures in the PDB for gp120, PDB ID: 3JWD was selected as being the one with the most full-length structure. 3JWD represents the structure of the HXB2 sequence simultaneously bound by CD4 and the 48D Fab. However, the 3JWD model, like most crystal structures, contains truncated regions representing the variable loops. Therefore, theoretical structural predictions of the full-length HXB2 gp120 molecule were made by applying the program CPHmodels 3.2 [46] (http://www.cbs.dtu.dk/services/CPHmodels/).

The quality of the predicted model was assessed by creating an alignment of the α-Carbon backbones of PDB ID: 3JWD and the prediction using the DaliLite server. [http://www.ebi.ac.uk/Tools/dalilite]. The CPH-model construct showed minor overall deviation through the core gp120 region with a root-mean-square deviation (RMSD) of 1.1 (Additional file 1: Figure S2). This model was used as a reference structure to determine patch regions.

Initial surface accessibility calculations were performed upon protein models with DSSP (http://mrs.cmbi.ru.nl/hsspsoap/). The ASA values from DSSP were then normalized according to the method of Singh et al. [33] (http://hoa.netasa.org) to calculate the relative surface area (RSA) where

RelativeSurfaceAreaRSA=AccessibleSurfaceAreaASAHighestObservedAreaHOA

The HOA values for each amino acid were derived in 2009 by Singh et al. [33] from an examination of the high quality structures present in the PDB. Surface accessible residues were determined to be those residues whose RSA was > = 20%.

Mean residue depth (DPR) is the mean distance between all of the atoms in a residue of interest and the nearest neighboring water molecule on the protein surface [39]. The mean residue depth for each gp120 model structure was calculated according to the method of Pintar et al. [39] using the tool provided at (http://hydra.icgeb.trieste.it/dpx) with a sphere radius of 1.4 Å.

In order to try and capture all of the key residues that may affect a competent antibody epitope, we evaluated “thick” surface patches. By combining RSA and DPR information during the patch identification process, we hope to capture a significant portion of the residue interaction network (RIN) [36,37] that influences residue orientation relative to the protein surface and have the potential of becoming eventually exposed themselves, given that proteins are dynamic structures and can change shape when bound to other proteins. To assure the inclusion of residues that may be part of the RIN, we examined patches within dimensions that make biological sense with respect to the size of an antibody footprint. We explored 3 different patch radii (8, 10, and 12 Å), as well as four different thresholds for maximum residue depth (0, 1.5, 2.5, and 3 Å). The patch lists were generated based on all these conditions -defined by RSA and DPR parameters-. The program generates a list of patch sequences, with each sequence consisting of named positions (e.g. N160) that are included in the patch. This patch list was then used in subsequent steps.

### Creation of patch vectors

In the next step, the matched phenotypes and genotypes of all viruses were formatted into patch vector files, with the patch topology established according to the predefined conditions (Figure 1A). For each antibody, a series of these patch vector files are generated, one file per patch. Each file contains the following for all viruses: the virus ID, IC_50_ measurement of the antibody under investigation, and a vector of binary (0 or 1) values representing mutations of the patch residues in that virus. The 0/1 vector is determined by comparing each position in the patch sequence to the reference; 0 if identical, 1 if different. Due to the high-dimensionality of the data, incorporating the individual amino acid changes was not computationally feasible. Additionally, since our goal was to identify key regions likely to be immunological hotspots, using the exact amino acid at each site as opposed to a binary (0/1) representation of the mutation wasn’t expected to make a significant difference in the outcome of the study.

### Data mining process

Using the patch vector files as input, we performed statistical and machine-learning techniques to correlate the neutralization response as measured by the IC50 (Additional file 1: Figure S1) with the genotype. We developed a boosted algorithm consisting of four multivariate and univariate models: multiple linear regression (MLR), logistic regression (LR), support vector machine (SVM), and Fisher’s exact test (FET). Data mining was performed in R [47] (http://www.R-project.org).

MLR was applied to the mutations in each patch captured as 0/1 vectors correlating the amino acid composition to the IC_50_ as a continuous measurement. We also developed 3 other models for correlating the neutralization response as a binary measurement with the patch vectors. We classified virus’ neutralization sensitivity against each antibody as response or no-response corresponding to IC_50_ < or > highest tested concentration, respectively (Additional file 1: Figure S1). Two multivariate models were built using LR and SVM techniques for performing classification in order to predict the antibody response. The SVM model was trained using libsvm in R package e1071 utilizing the linear kernel, 20-fold cross-validation, and cost = 1 [48] (R package version 1.5-26). Fisher’s exact test was also utilized as a univariate analysis calculating odds-ratio and statistical significance (p-value) to identify strong associations between presence or absence of a mutation with the neutralization response.

These data mining models were applied to all sets of patch vectors, each set corresponding to a particular antibody. Patches that included statistically significant (p-value < 0.05) amino acid sites by MLR, LR, or FET, or achieved an overall accuracy of >70% by SVM cross-validation, were marked as significant and were considered for further evaluation. Notably, no correction for multiple testing was performed, as the goal was to be inclusive for discovering the most potential antibody targets. Only patches identified as significant by all four methods were used, with the hypothesis that patches with highest agreement by multiple models would be more likely to be substantially affecting the neutralization sensitivity, and therefore, more probable to be part of an epitope or epitope network.

In each significant patch, residues with significant correlation with the neutralization response identified as p-value < 0.05 were highlighted and stored as regions that are impactful with regards to the neutralization response to the antibody.

### Identification of epitope networks

We examined a set of conditions for patch composition, as defined by the particular patch radius and thickness. Patch radii 8, 10, and 12 Å in combination with depths of 0, 1.5, 2.5, and 3 Å were investigated. We then selected the condition that included residues previously reported to be part of known antibody epitopes [3,4,27,42] and by eliminating conditions that resulted in redundant patch clusters. Patches were generated based on the selected condition, and evaluated by the four data mining models. We obtained patches determined to be significant by all four models, i.e. patches that contain statistically significant residues by MLR, LR, and FET, and high concordance of the neutralization activity with the predicted response by SVM. To identify frequently occurring clusters of residues in these key patches which are likely to be important in antibody binding as a network, the frequency of occurrence of each residue across the significant patches was obtained (Figure 1B). We noticed that frequencies were not consistently high or low for different antibodies, and that the highest observed frequency varied considerably amongst these patches. Thus, in order to allow a consistent comparison across all patch sets for different antibodies, the frequency of each patch cluster was normalized by the frequency of the most commonly occurring residue (normalized frequency = frequency/max(frequency)). Finally, the residues occurring with an above average normalized frequency (>50%) in the significant patches were identified as epitope network candidates for the particular antibody.

### Structural mapping of potential epitopes

In order to visualize the spatial organization of the amino acids composing an epitope network, residues were mapped onto the surface of the CPH-model of gp120 protein monomer using Chimera [49]. Given the unique quaternary structure of gp120, residues were also modeled onto a theoretical gp120 trimer structure to provide a method for evaluating candidate epitope networks arising from potential protein-protein interfaces. Predicted epitope networks were then visually evaluated through comparison with known epitopes or superimposed upon other gp120 crystal structures (PDB ID: 2NY7) which contained additional interacting molecules such as the b12 fab and CD4 to evaluate the accuracy of the epitope network prediction.

### Evaluation of the results

In order to evaluate the validity of the predicted epitope networks, site-directed mutagenesis (SDM) experiments were performed. Mutations were introduced using two steps polymerase chain reaction (PCR). In the first step, the desired mutation is introduced by a PCR primer used to amplify one part of the target gene. In the second step, this PCR product is used as a megaprimer to amplify the full gene containing the mutation. All mutations were confirmed by sequencing. Since the goal was to interrogate genotypic reasons for IC50 differences within viruses that are more likely to be circulating naturally, we used selected clones from our virus panel as the backbone. We compared the sequences of the clones from a single infected individual that exhibited different IC50 responses to one or more mAbs. We then selected clones from the same donor where the sequence differences overlapped, at least partly, with the predicted epitopes for that mAb. There were other sites that differed between the clones that were outside of the predicted epitopes. The SDM experiments were designed to introduce mutations at the sites that were different between the clones and were within the predicted epitopes. We wanted to investigate whether neutralization sensitivity can be restored in a resistant clone, or reverted back in a sensitive clone by introducing mutations at these selected sites. We ran these SDMs using Monogram’s neutralization assay [9], and obtained IC50 values against the mAbs under investigation.

Additionally, performance of the THIPA method was evaluated by developing a prediction model based on the subset of the residues that were identified to be significant by all four data mining models. We built an SVM model using only these significant sites as input to predict neutralization sensitivity to each antibody. We then examined its overall accuracy by comparing the predicted response to the measured antibody titer classified into positive or negative, corresponding to IC_50_ > or < max tested antibody concentration, respectively (Additional file 1: Figure S1). We obtained sensitivity (true positive rate) and specificity (true negative rate) for detection of resistance to the particular antibody. In order to assure that the viral subtype doesn’t impose a bias in terms of performance characteristics of the algorithm, we also examined the accuracy of the predictions within different subtypes. To make sure we have sufficient sample size for this analysis, only subtypes with at least 10 samples were included (A, AE, AG, B, C, D, F1, and G). Additionally, we studied previously published epitopes [4,27] and compared those with the identified epitope networks by the THIPA process.

## Results

In this paper, we present a novel method called thick patch analysis (THIPA) for predicting antibody epitope networks by correlating neutralization phenotype to the genotype and the antigen structure. We define an epitope network as a patch of residues that are antigenic determinants and have significant association with the neutralization response. Neutralization sensitivity for 21 HIV-1 monoclonal antibodies (b12, PG9,16, PGT121 - 128, PGT130 - 131, PGT135 - 137, PGT141 - 145, and PGV04) was determined by Monogram’s neutralization assay [9] and was captured as IC_50_ titers against a panel of 282 clonal viruses. Viral gp120 sequences were mapped onto structural patches and were then correlated with the neutralization response of these antibodies. Using data mining techniques, we identified residues that significantly impact neutralization activity, and are therefore considered to be potential antibody targets. For validating this method, we performed site-directed mutagenesis (SDM) experiments using the residues predicted to be part of the epitope network of multiple nAbs. Additionally, we used the identified set of significant residues as input, and built SVM models to perform prediction of the neutralization response, and evaluated the accuracy of the predictions. The predicted epitope networks were also compared with the available literature on PG9, PG16 and PGV04.

### Predicted epitopes

The pipeline generated 268 unique patches on the gp120 surface using the theoretical model structure as a Template. A number of these patches were found to be significant, as identified by >70% accuracy by SVM or containing residues that are significantly associated with the neutralization response by MLR, LR, or FET. Several of these significant patches were identified by all four models, and were considered to be highly impactful. The number of significant patches varied depending on the monoclonal antibody (mAb). Some of these mAbs could be grouped into families as they originated from the same donor: PG-9 and −16, PGT-121-131 135–137, and PGT-141-145 [40,41].

**Figure 2:**
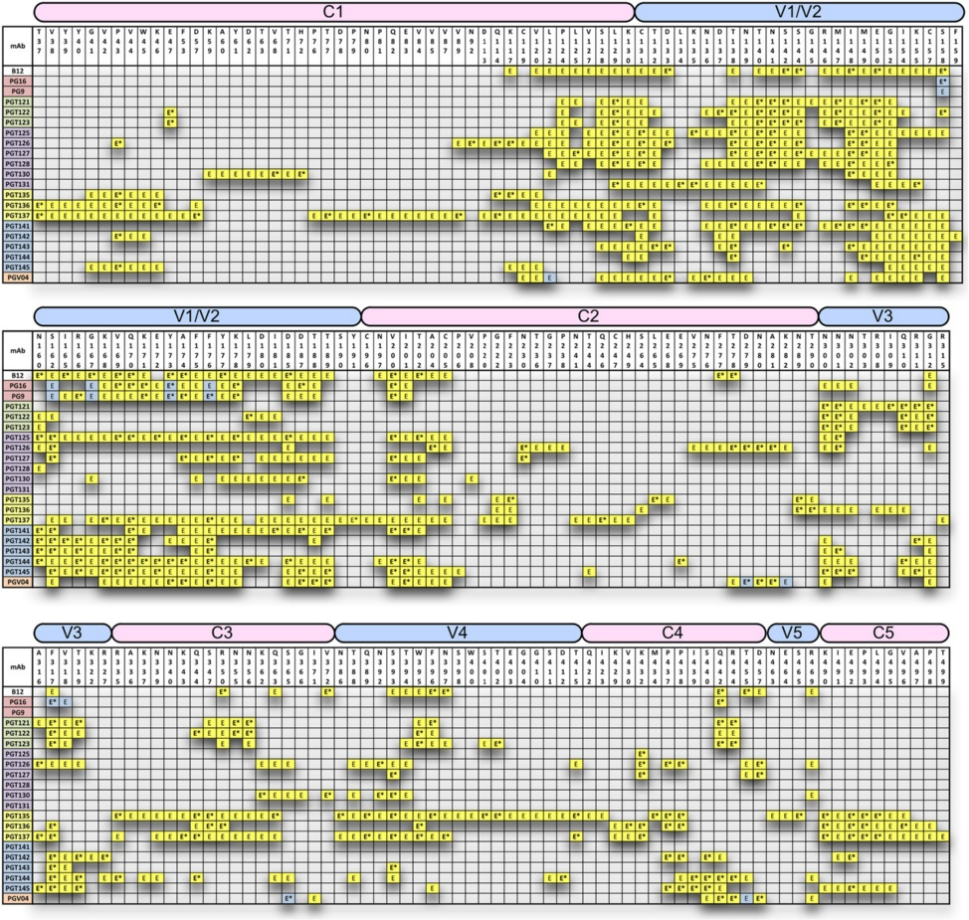
**Predicted Epitope Network Residues for all mAbs.** The amino-acid composition of the predicted epitope networks is noted as the prefix of the position on top of each array (for example, N160 indicates Asparagine at position 160 of gp120). Residues marked with “E” are predicted to be part of the antibody epitope network. The residues that are highly significantly associated with antibody response (MLR p-value <0.001) are marked by *. Residues reported by Walker et al. to impact PG9 and PG16 sensitivity [4] and those identified by Falkowska et al. for PGV04 [27] are color-coded in blue.

We investigated the results across several patch depths and thicknesses, and selected an optimal condition by examining for coverage and redundancy as well as taking the theoretical size of an antibody footprint into account. Patch depths 1.5, 2.5, and 3 Å generated very similar compositions of residues, and depth = 0 Å included no known epitope residues. We selected radius = 12 Å and depth = 2.5 Å as the optimal condition, and are reporting the final results retrieved based on this condition. The predicted epitope networks were spatially oriented into overlapping or neighboring clusters of residues, and shown in Figure 2. Remarkably, among all significant patches for these mAbs, the most frequently occurring residues were predominantly located within the variable loops V1/V2 of gp120. This finding is consistent with previously identified epitopes, in particular for PG-9 and −16 [4,50,51].

**Figure 3:**
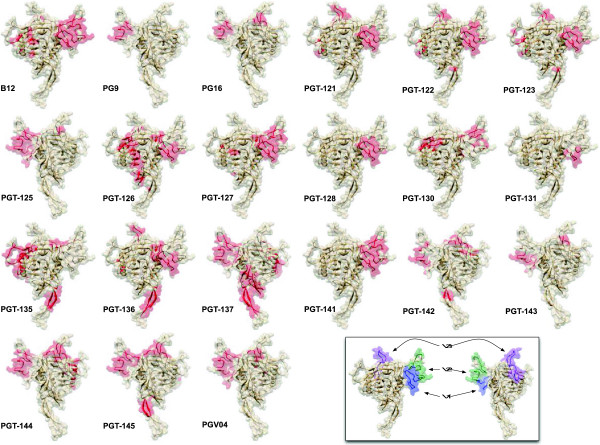
**Predicted Epitope Networks for all mAbs in 3D.** Predicted epitope networks are indicated in red for each antibody. The 3D models represent a theoretical view of full-length HXB2 gp120 monomer by the CPHmodels program [46]. Model is rotated for each antibody to show the best view of network residues. Visualizations were generated using UCSF Chimera [49]. INSET: General location of the variable regions V1, V2, and V3 are indicated. Notably, the most overlapping residues between the predicted epitope networks of different mAbs were predominantly located within the variable loops V1/V2 of gp120.

**Figure 4:**
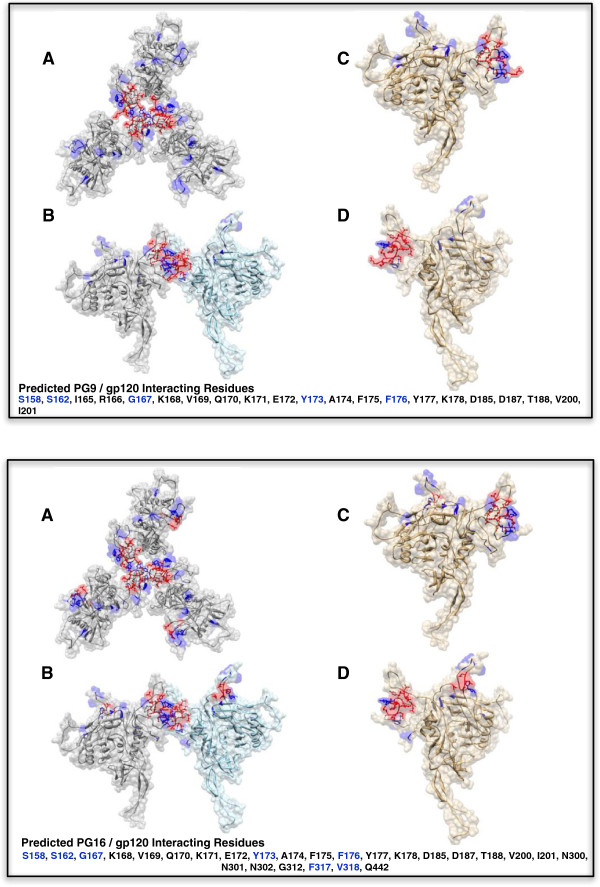
**Predicted Epitope Network Residues vs. Published Epitopes for PG9 (top panel) and PG16 (bottom panel).** Residues predicted to be part of an interacting network are represented by ball-and-stick and are colored red. Residues reported by Walker et al. to impact PG9 and PG16 sensitivity [4] are colored blue. Overlap between predicted and published residues is indicated by blue residues which are also shown in ball-and-stick representation. The 3D models represent a theoretical view of full-length HXB2 gp120 as generated by the CPHmodels program [46]. **A** and **B** depict a hypothetical trimer seen from the top and side respectively, while **C** and **D** represent a monomer representation seen from the front and back respectively. Correct orientation of trimer subunits was obtained by aligning the Cα backbone of the predicted models with the corresponding chains of the trimer structure (PDB ID: 2NY7). Visualizations were generated using UCSF Chimera [49].

Subsequently, the identified epitope network residues based on the optimized condition were mapped onto the theoretical full-length gp120 CPH-model, and displayed in Figure 3. Notably, when the predicted epitope networks for PG9 and PG16 were modeled on the surface of the CPH-model structure together with the published residues [4], we observed considerable overlap as shown in Figure 4. This demonstrates that the THIPA method can be utilized to narrow down the search for antibody epitopes into key regions that are most likely to be involved in antibody binding, both directly in an epitope and indirectly as affecting epitope shape or function. These epitope networks are then candidates for confirmation by site-directed mutagenesis to identify potential vaccine immunogen constructs.

### Site-directed mutagenesis experiments

**Figure 5:**
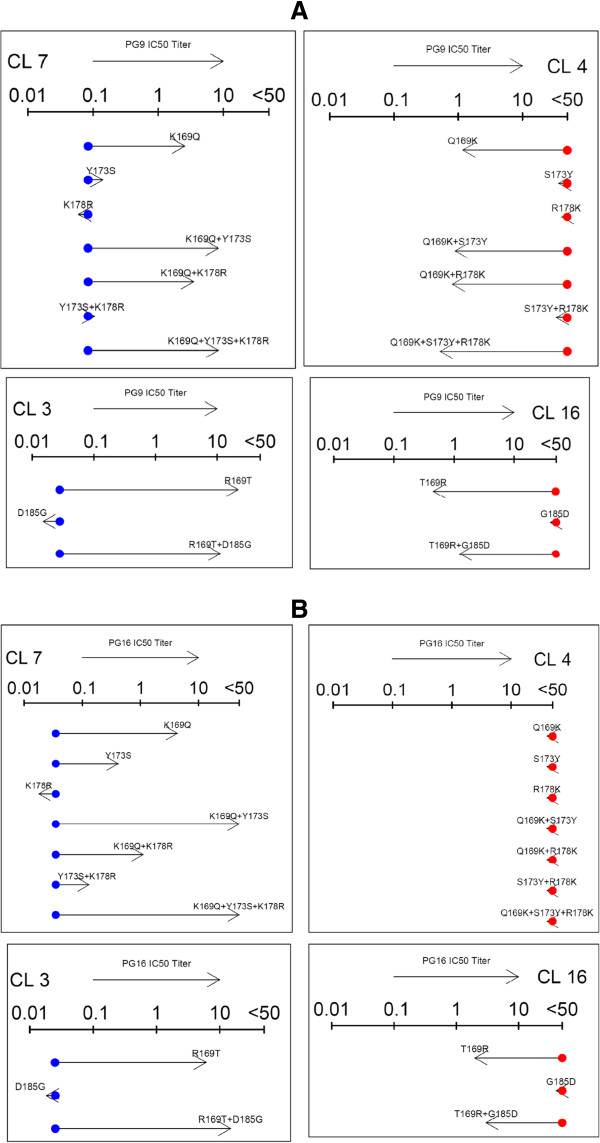
**Site-Directed Mutagenesis:** PG9 **(A)** and PG16 **(B)** IC50 titers for clones after introducing mutations into sites identified to be part of the predicted epitope networks. The dots show the IC50 of the original clone, and the arrows show the IC50 of the mutant. Red dots and higher IC50 measurements indicate reduced sensitivity, and green dots and lower IC50 titers increased sensitivity. Clones 4 and 7 (CL4 and CL7) are from the same donor, as well as clones 16 and 3 (CL16 and CL3).

We selected a subset of the residues within the predicted epitope networks that were identified for PG9 and PG16, and performed site-directed mutagenesis (SDM) experiments using clones that differed in these positions. We used a resistant or sensitive clone as the backbone, and examined if sensitivity can be restored or reduced, respectively, by introducing mutations at the sites of the predicted epitope networks. Clone pairs 4 and 7 (CL4 and CL7) as well as 16 and 3 (CL16 and CL3) that are from the same donor were selected. CL4 and CL16 had resistant IC50 titers against PG9 and PG16, and CL7 and CL3 were sensitive (Figure 5, Additional file 1: Table S1). Notably, these selected clones had no mutation at some of the signature sites such as positions 156–162 [4].

As displayed in Figure 5 and Additional file 1: Table S1, sensitivity of CL4 to PG9 could be restored by introducing a Q-to-K mutation at position 169 (numbering provided based on the gp120 of HXB2 reference). Sensitivity could be further enhanced by additional mutations S173Y and R178K. No increase in the neutralization sensitivity to PG16 was observed with these mutations. We observed a major loss of the neutralization sensitivity to PG9 and PG16 in CL7 when introducing the reverse mutation K169Q, as well as in combination with Y173S and K178R. CL3 which was sensitive to both PG9 and PG16 became less sensitive with the introduction of mutations R169T and R169T + D185G. On the flip side, the resistant clone CL16 gained sensitivity to PG9 and PG16 with the reverse mutations T169R and T169R + G185D. Notably, 185 as a single mutation had no significant impact on the IC50 titers against PG9 and PG16, however, as shown in Table S1, response to b12 was positively impacted by D185G and negatively by reverse mutation G185D; 185 was indeed part of the predicted epitope network of b12.

Since these positions were predicted to be part of the epitope networks for a big group of the mAbs (in particular, 169 is predicted for b12, PG9, PG16, PGT125, PGT137, PGT142-145, as well as PGV04), IC50 titers of these SDMs against all the mAbs under investigation are shown in Table S1. Though, we did not expect that these particular SDMs would necessarily impact sensitivity against other mAbs, since the subset of sites selected for interrogation was based on the predicted networks for PG9 and PG16. Thus, the possibility of other differing sites between the clones that happen to be critical for other mAbs could not be eliminated.

### Performance evaluation

We obtained performance characteristics for the THIPA method by building predictive models based only on the significant residues identified by all four models, and assessed the accuracy as the ability to correctly predict neutralization response. We also examined concordance of the predictions with the neutralization sensitivity within each subtype. We obtained concordance rate within a subtype as the number of true positive and negative samples divided by total, and calculated the standard-deviation between accuracy measurements across the different subtypes. These results are shown in Table 1. In general, concordance of the neutralization sensitivity with the predicted response by the algorithm as obtained from the SVM cross-validation accuracy was high (>80%) for all antibodies. This suggests that the THIPA process could successfully identify significant residues associated with antibody binding, since only a small subset of all residues was used as SVM input and not the whole sequence. Notably, this high accuracy was maintained within all different subtype groups, as demonstrated by the narrow range of standard-deviations [2.42 - 10.35].

**Table 1:** Performance evaluation

	**Total number of responders and non-responders to the antibody**		**Performance in the full dataset**					**% accuracy within subtypes**								**Standard deviation across subtypes**
**Antibody**	**# Responders**	**# Non-Reponders**	**Sensitivity (%)**	**Specificity (%)**	**Accuracy (%)**	**FN**	**FP**	**A (N = 21)**	**AE (N = 24)**	**AG (N = 19)**	**B (N = 55)**	**C (N = 32)**	**D (N = 55)**	**F1 (N = 24)**	**G (N = 41)**	
b12	61	221	98.2	77	93.6	4	14	90.5	91.7	89.5	92.7	96.9	94.5	100	92.7	3.47
pg16	177	105	74.3	93.2	86.2	27	12	90.5	83.3	94.7	89.1	75	89.1	83.3	82.9	6.10
pg9	178	104	64.4	94.4	83.3	37	10	85.7	91.7	94.7	87.3	71.9	81.8	83.3	75.6	7.65
pgt121	165	117	88	97.6	93.6	14	4	100	100	100	90.9	90.6	90.9	91.7	95.1	4.45
pgt122	150	132	88.6	92	90.4	15	12	95.2	100	89.5	90.9	90.6	85.5	83.3	92.7	5.26
pgt123	159	123	82.9	93.1	88.7	21	11	85.7	100	84.2	89.1	93.8	87.3	83.3	87.8	5.54
pgt125	130	152	90.8	82.3	86.9	14	23	76.2	87.5	89.5	90.9	87.5	92.7	91.7	78	6.25
pgt126	149	133	89.5	79.2	84	14	31	85.7	91.7	78.9	83.6	78.1	87.3	91.7	78	5.71
pgt127	118	164	87.2	82.2	85.1	21	21	90.5	87.5	94.7	80	78.1	90.9	87.5	82.9	5.74
pgt128	173	109	64.2	92.5	81.6	39	13	81	66.7	94.7	83.6	87.5	89.1	70.8	70.7	10.11
pgt130	146	136	82.4	87.7	85.1	24	18	81	87.5	78.9	85.5	84.4	87.3	87.5	82.9	3.23
pgt131	101	181	90.6	76.2	85.5	17	24	85.7	79.2	78.9	89.1	96.9	80	87.5	80.5	6.33
pgt135	66	216	98.1	77.3	93.3	4	15	95.2	100	89.5	98.2	87.5	94.5	87.5	92.7	4.72
pgt136	31	251	99.2	77.4	96.8	2	7	100	100	94.7	98.2	90.6	98.2	100	92.7	3.67
pgt137	31	251	100	61.3	95.7	0	12	100	100	94.7	94.5	93.8	96.4	95.8	95.1	2.42
pgt141	121	161	92.5	90.1	91.5	12	12	66.7	95.8	84.2	94.5	93.8	98.2	83.3	92.7	10.35
pgt142	139	143	90.9	89.9	90.4	13	14	100	83.3	89.5	87.3	90.6	90.9	91.7	95.1	4.98
pgt143	140	142	89.4	92.9	91.1	15	10	95.2	79.2	94.7	92.7	84.4	87.3	95.8	100	6.91
pgt144	73	209	96.7	86.3	94	7	10	100	91.7	94.7	92.7	93.8	96.4	87.5	95.1	3.64
pgt145	132	150	89.3	91.7	90.4	16	11	85.7	87.5	84.2	90.9	93.8	90.9	91.7	95.1	3.84
pgv04	216	66	72.7	95.4	90.1	18	10	85.7	95.8	84.2	94.5	90.6	89.1	95.8	82.9	5.22

Comparing the predicted neutralization sensitivity of the SVM models using only the significant sites within gp120 as input, to the measured antibody response. We obtained sensitivity and specificity for detection of resistance to the particular antibody. Overall accuracy of the predictions in the whole dataset as well as within each subtype was determined. Standard-deviation of % accuracy across those subtypes is shown in the last column.

## Discussion

We describe here a novel multi-modal method for predicting antibody-specific epitope networks, utilizing antigen structure models, viral genotypes, neutralization assay results, and data mining techniques, together in a pipeline called thick patch analysis (THIPA). The goal of this study was to identify epitope networks of HIV-1 broadly neutralizing antibodies (nAbs). The predicted epitope networks are clusters of antigenic residues significantly associated with binding and neutralization activity. We aimed to identify both conformational and linear epitopes by systematic mapping of the genotypic information onto 3D structural models and correlating that with the neutralization phenotype. For this purpose, we utilized the IC50 measurements of 21 monoclonal antibodies against a panel of 282 clonal HIV-1 viruses of various subtypes, co-receptor tropism, and neutralization sensitivity. Patches with a particular thickness and radius were identified on the HXB2 gp120 theoretical structure, and the genotype of each virus was mapped onto these models. The viral sequence located in each patch was captured as a binary (0/1) vector, representing presence (1) or absence (0) of a mutation as compared to HXB2. We then correlated these vectors with the IC_50_ data by performing multiple statistical and machine-learning methods, and identified residues and patches significantly associated with the neutralization response to each antibody. Finally, we evaluated the significant patches detected by the data mining tools, and predicted epitope networks for HIV-1 monoclonal antibodies (mAbs).

The THIPA pipeline attempts to identify regions within the viral envelope that are likely to be targeted by nAbs, and therefore are critical to be studied for successful vaccine design. This computational method scans the gp120 molecule through patches with a probable size for an antibody footprint, and as such, it not only allows the examination to be applied in a structurally meaningful way, but also narrows down the search dimension to smaller area that is more computationally feasible to survey. The process utilizes sequences of naturally circulating viruses, rather than lab strains, which are more likely to be exposed to nAbs and thus, could elicit their escape mechanism. Additionally, this method allows the study of genotypic patterns and their impact on neutralization in context of co-occurring mutations [52]. One of the major data components included in this method is derived from a cell-based neutralization assay that determines the phenotype of the antibody in presence of a given virus. Consequently, residues that are identified as significant can elicit mechanisms of viral escape from the immune response. Another key aspect in the design of this method is the implementation of a boosted technique, which utilizes an ensemble of statistical and machine-learning models, each with unique set of strengths, allowing us to evaluate the network of information from different angles. Notably, since our purpose of applying these models was to discover potential immunological hotspots, and not to build a predictive algorithm, no fine-tuning of the model parameters was necessary. In particular, SVM accuracy cutoff of 70% using a linear kernel and default parameters allowed successful identification of significant patches without further parameter adjustment.

In order to examine the validity of our results and ensure that the identified residues are in-fact related to the neutralization activity, we performed site-directed mutagenesis (SDM) experiments using clones that differed in the residues identified to be part of the predicted epitope networks of a group of mAbs. The SDM results confirmed significant associations between a set of residues we investigated and the antibody response, suggesting that the THIPA method was able to correctly identify key residues out of many mutations. This is also remarkable given that there were many other sites that differed between the clones. The inclusion of mutation 169R in the envelope protein of a neutralization-resistant virus greatly increased the sensitivity to PG9 and PG16, suggesting that the normally shielded regions of the protein were made accessible to the antibody by this change. The mutation 169 K resulted in an either enhanced or reduced susceptibility to a number of mAbs. The reverse mutations K169Q and Y173S had much more dramatic effect on the neutralization activity against PG9 and PG16 mAbs than the Q169K and S173Y mutants. This might be due to the presence of Glutamine and Serine that help shield the neutralizing epitopes in the specific backbone. Although positions 178 and 185 were predicted to be significant for a group of mAbs, introducing mutations at these sites didn’t affect the IC50 titers significantly suggesting that additional sites have to be involved in the antibody binding. One possibility is that multiple amino acid changes might modify the overall shape of the envelope protein, thus exposing normally hidden regions in the HIV-1 envelope protein to the antibodies. Additional experimental studies would be required in order to confirm the impact of the newly identified residues.

For validating our results, we also developed computational models based only on the subset of significant residues. As shown by the high predictive accuracy of these models in Table 1, the identified residues were found to substantially impact the neutralization sensitivity of the respective antibody. Predictive accuracy was also shown to be high across the different subtype groups, suggesting that subtype was not a confounder with respect to the quality of the predictions.

Additionally, we evaluated the structural implications of our results and compared them with the published literature. We demonstrated that this method can accurately identify previously published epitope regions of three HIV-1 mAbs (PG9, PG16, and PGV04), and provide information on other regions that may contribute to neutralization response or the corresponding escape mechanism. The predicted epitope network residues for PG9 and PG16 together with their published epitopes depicted on the trimer structure are shown in Figure 4. The trimer view is provided since both nAbs PG9 and PG16 belong to the family of quaternary-structure-specific antibodies [53].

It should be noted that the structural component of the THIPA process is fairly robust with regard to the structural model used. We compared the patch lists derived from the truncated gp120 monomer structure (PDB ID: 3JWD) and those derived from our full length predicted monomer structure (data not shown). Excluding sequence regions that are clipped in the 3JWD structure, there was a high degree of agreement in the patch definitions. We would predict that the differences in specifics of loop structure prediction would not significantly change patch definitions because they would always be defined as surface residues. However, if two core structures with different conformations were used (e.g. prefusion gp120 vs . postfusion gp120), then we would predict some unique patches would arise from that and potentially change epitope network prediction outcomes slightly.

Since the goal of this process was to maximize the ability to detect disparate potential antigen binding sites, it is critical to use diverse viruses with naturally occurring variants. We used 282 clones that differ widely in neutralization sensitivity, co-receptor tropism, and subtype. However, there remain residues that are conserved across all clones, so their impact as antigenic determinants could not be evaluated. Accordingly, a caveat of utilizing a limited set of viruses for the THIPA method is that the data mining processes are unable to find potential epitope network residues if there are no mutations in any of the sequences at that particular position. For example, b12 and PGV04 are known to rely on making a salt-bridge with D368 [54] but that position is completely conserved in our virus panel and therefore could not be identified for either antibody. We recognize that our method is relevant for identifying highly variable regions associated with antibody neutralization (Additional file 1: Figure S3). Another limitation of this method is in detecting binding sites where low level mutations can be tolerated by the antibody, and therefore, may not be associated with a significant change in IC_50_.

There may be other viral properties, such as number of glycosylation sites [42], that can impact the neutralization response and haven’t yet been explored by this method. Including a broad spectrum of viral sequences and all possible genotypic determinants into the analysis is likely to increase the accuracy of the results. Also noteworthy is the fact that we used a theoretical model in our study in order to overcome the barrier of truncated variable loops in the available x-ray structures. Although the chosen model had minor deviations from the actual crystal structure (Additional file 1: Figure S2), and it is a reasonable assumption that the variable loops can be flexible in space and thus not requiring to conform to a fixed structure, it will be beneficial and interesting to repeat the study using full-length crystal structures as they become available. An example of the risk associated with using a theoretical model is N332 for PGT121 which was reported by Julien et al. [55]. In the theoretical model, the angle of the N332 is rotated inward and only the tip of the residue presents a surface, so the site didn’t make the depth cutoff in our method. Notably, other regions do overlap between our predictions and the reported PGT121 epitope, particularly regions in V3, for instance N301.

## Conclusions

Despite the limitations, the thick patch analysis method can provide a useful tool to rapidly and automatically scan protein structures and identify epitope network regions associated with neutralization response. Our novel *in-silico* prediction method narrows down the search space for immunological hotspots to key variable regions. These regions are then good candidates for further evaluation by experimental studies as HIV-1 neutralizing antibody epitopes. Furthermore, this method can be adapted into other disease areas, particularly for predicting epitopes of highly variable viruses.

## Competing interests

All authors have received salary from Monogram Biosciences and its parent company, LabCorp, in the past 2 years. PP, CJP, TW, and MH own LabCorp stock. CJP is a VP and Officer of LabCorp.

## Authors’ contributions

MH designed the study, performed statistical and machine learning analysis, and wrote the manuscript. MCE performed computational, sequence, and structural analysis, and drafted the manuscript. ACP performed computational and statistical analysis. TW, PP, and AP performed the phenotypic and genotypic experiments. CJP and TW designed and managed the lab experiments. All authors read and approved the final manuscript.

## Supplementary Material

Additional file 1: Figure S1 IC50 Curves: neutralization sensitivity is classified into response (e.g. the right curve) or no-response (e.g. the left curve) corresponding to IC_50_ < or > highest tested concentration, respectively. **Figure S2**: Actual versus Theoretical Structure: PDB ID: 3JWD (actual) and CPH (theoretical) structure models are shown. Truncated regions in the actual model are circled by yellow dotted line. **Figure S3**: Amino-Acid Variability in the Clonal Sequences for PG9 and PG16 Epitope Network Sites: residues identified within PG9 and PG16 epitope network can be strongly variable. Observed amino acids at these sites are displayed as a color-coded bar chart, with every color corresponding to one amino acid according to the left legend. Insertion is marked as “Z” and deletion is marked as “^”. **Table S1**: Site-Directed Mutagenesis: IC50 titers against 21 mAbs after introducing mutations into sites identified to be part of the predicted epitope networks. Clones 4 and 7 (CL4 and CL7) are from the same donor, as well as clones 16 and 3 (CL16 and CL3). Click here for file
