# Supplementary material for: Predicting HIV-1 broadly neutralizing antibody epitope networks using neutralization titers and a novel computational method
Source: BMC Bioinformatics. 2014 Mar 19;15:77. doi: 10.1186/1471-2105-15-77 (PMC3999910; doi:10.1186/1471-2105-15-77)
Supplement: Additional file 1: Figure S1 — IC50 Curves: neutralization sensitivity is classified into response (e.g. the right curve) or no-response (e.g. the left curve) corresponding to IC50 < or > highest tested concentration, respectively. Figure S2: Actual versus Theoretical Structure: PDB ID: 3JWD (actual) and CPH (theoretical) structure models are shown. Truncated regions in the actual model are circled by yellow dotted line. Figure S3: Amino-Acid Variability in the Clonal Sequences for PG9 and PG16 Epitope Network Sites: residues identified within PG9 and PG16 epitope network can be strongly variable. Observed amino acids at these sites are displayed as a color-coded bar chart, with every color corresponding to one amino acid according to the left legend. Insertion is marked as “Z” and deletion is marked as “^”. Table S1: Site-Directed Mutagenesis: IC50 titers against 21 mAbs after introducing mutations into sites identified to be part of the predicted epitope networks. Clones 4 and 7 (CL4 and CL7) are from the same donor, as well as clones 16 and 3 (CL16 and CL3). [file 1471-2105-15-77-S1.doc]

Supplemental Figures/Tables.

**Predicting HIV-1 Broadly Neutralizing Antibody Epitope Networks Using Neutralization Titers and a Novel Computational Method**

Mark C. Evans, Pham Phung, Agnes C. Paquet, Anvi Parikh, Chris J. Petropoulos, Terri Wrin, Mojgan Haddad*

Monogram Biosciences Inc., South San Francisco, CA, USA

*Corresponding author:

Mojgan Haddad

Monogram Biosciences

345 Oyster Point Blvd., South San Francisco, CA 94080

Phone: (650) 616-3645

Fax: (650) 616-3652


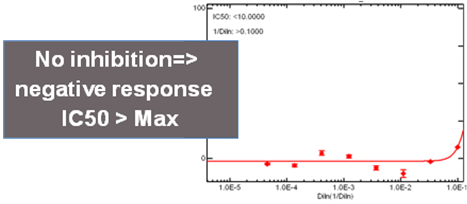

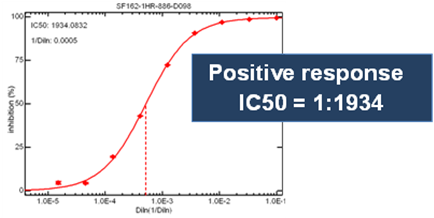


**Figure S1.**


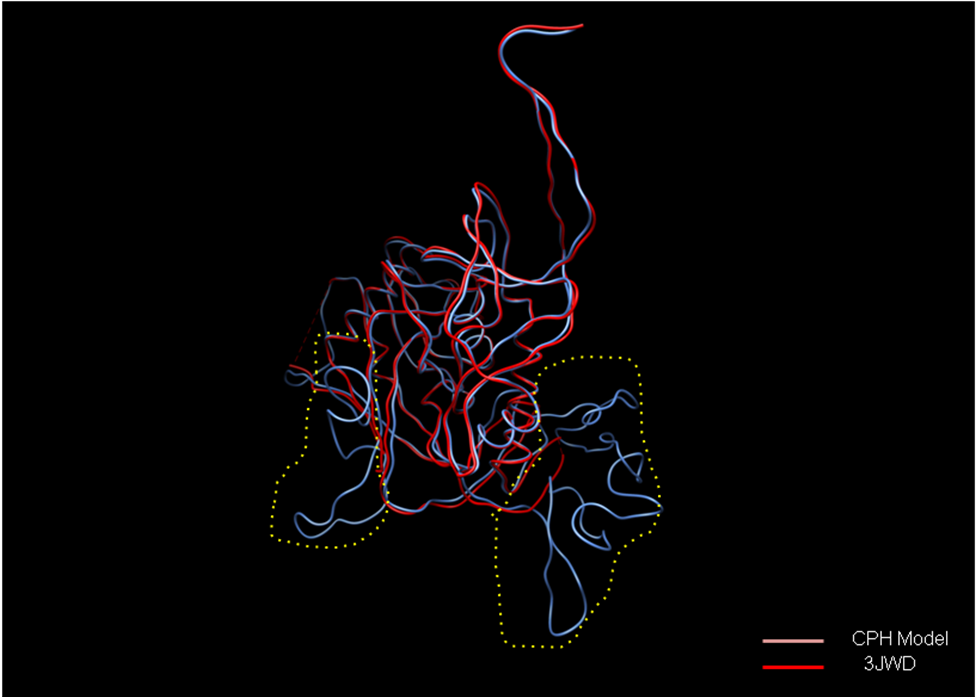


**Figure S2.**


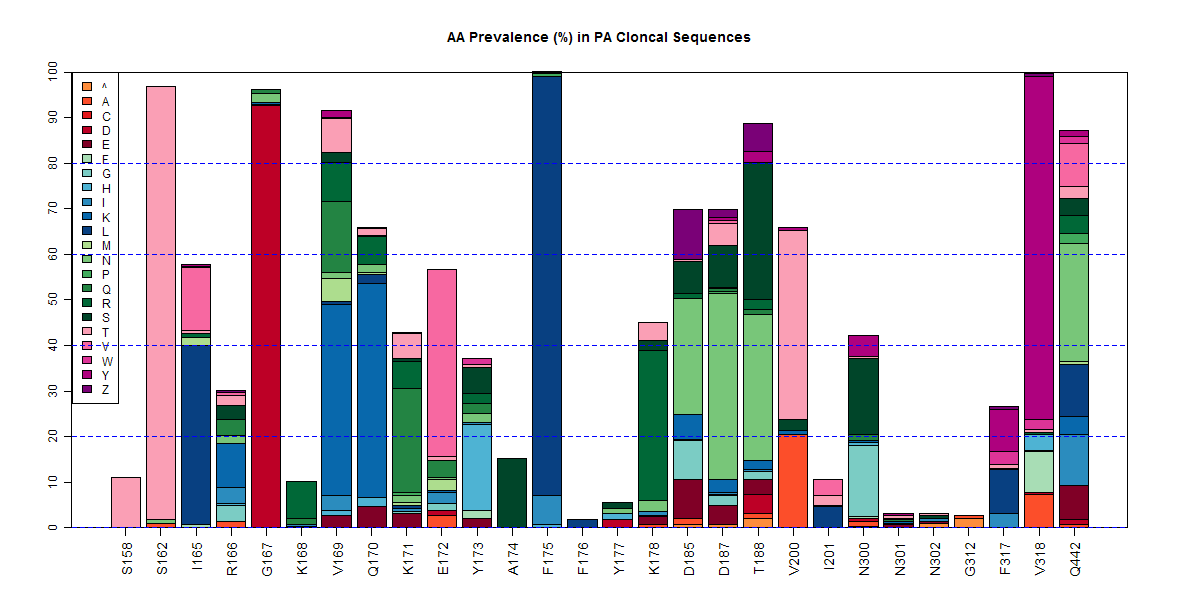


**Figure S3.**


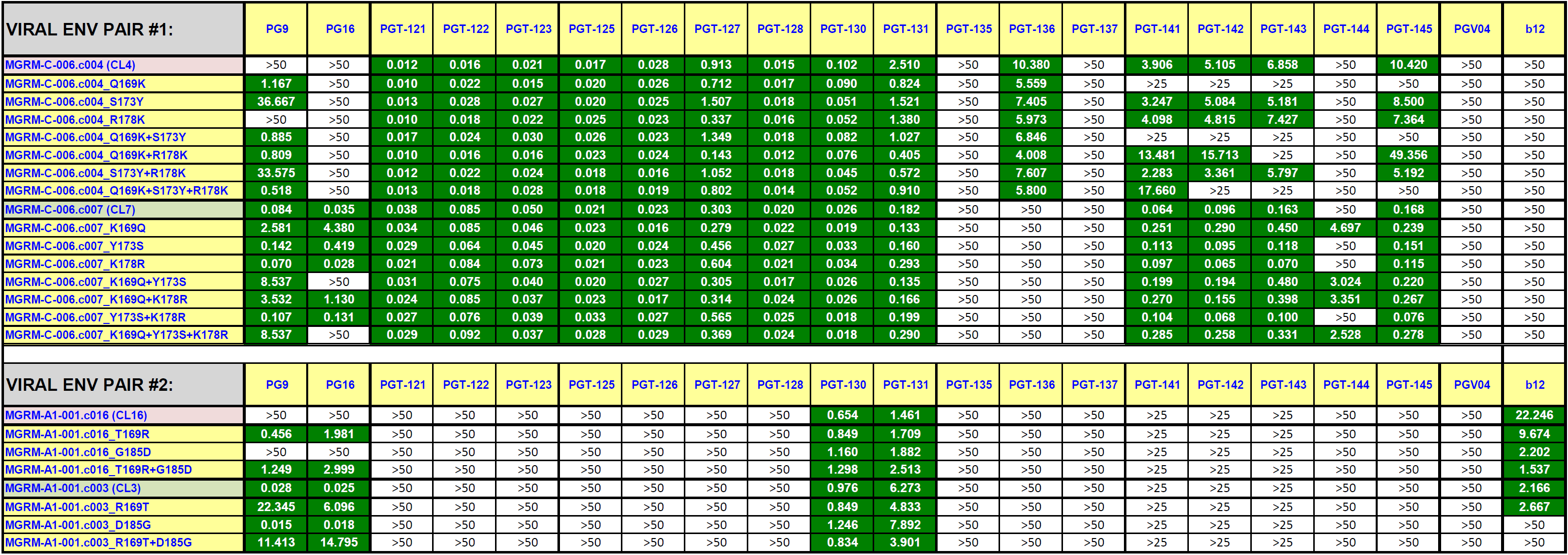


**Table S1.**
